# A systematic review and meta-analysis of cross-sectional studies examining the relationship between mobility and cognition in healthy older adults

**DOI:** 10.1016/j.gaitpost.2016.08.028

**Published:** 2016-10

**Authors:** Naiara Demnitz, Patrick Esser, Helen Dawes, Vyara Valkanova, Heidi Johansen-Berg, Klaus P. Ebmeier, Claire Sexton

**Affiliations:** a FMRIB Centre, Nuffield Department of Clinical Neurosciences, John Radcliffe Hospital, University of Oxford, OX3 9DU, UK; b Movement Science Group, Oxford Brookes University, OX3 0BP, UK; c Department of Psychiatry, Warneford Hospital, University of Oxford, OX3 7JX, UK

**Keywords:** Gait, Balance, Memory, Processing speed, Executive function, Healthy ageing

## Abstract

Ageing is associated with declines in cognitive function and mobility. The extent to which this relationship encompasses the subdomains of cognition and mobility remains unclear, however. We searched MEDLINE and EMBASE databases for cross-sectional studies examining the association between objective mobility measures (gait, lower-extremity function, balance) and cognitive function (global, executive function, memory, processing speed) in healthy older adults. Of the 642 studies identified, 26 studies met the inclusion criteria, with a total of 26,355 participants. For each feature of physical mobility, the relation to each aspect of cognition was reviewed. In the context of each association, we summarised the results to date and performed random-effects meta-analyses of published data. Reviewed findings suggest that individuals with better mobility perform better on assessments of global cognition, executive function, memory and processing speed. Not all measures of mobility were equally associated with cognitive function, however. Although there was a larger number of gait and lower-extremity function studies, and this may have driven findings, most studies examining balance and cognition measures reported no significant results. Meta-analyses on reported associations supported results by revealing significant, albeit small, effect sizes in favour of a positive association between performance on mobility measures and cognitive assessments. Future research should aim to establish the mechanisms driving this relationship, as this may identify predictors of age-related impairments.

## Highlights

• Twenty-six studies were identified as eligible for this systematic review.
• Mobility was positively associated with cognitive measures in healthy older adults.
• The cognition-mobility relationship spans across cognitive domains.
• Meta-analyses on extracted data revealed significant, albeit small, effect sizes.

## 1 Introduction

With a rapidly growing older population, identifying modifiable factors that can contribute to healthy ageing is a public health priority. Mounting evidence has highlighted the importance of maintaining physical mobility in old age. Unfortunately, this is a challenging task given mobility impairments are extremely common in the ageing population [1]. Poor mobility can lead to a cascade of other detrimental factors such as fear of going out, increased social isolation, poor quality of life, and hospitalisations [2], [3]. Moreover, there is evidence to suggest that poor mobility may be associated with poor cognitive function [4], [5]. Establishing such relationships is important; if associations between mobility and cognition are found this provides a clear rationale for assessing both cognitive and mobility outcomes in interventions targeting either domain, and also argues for developing combination interventions that jointly target both domains.

Both mobility and cognition are umbrella terms that span across multiple measurement domains. Mobility, for example, involves walking through diverse environments, maintaining balance whilst doing so, and being able to rise from beds and chairs. Epidemiological studies have shown that measures of gait, balance and chair rises are predictive of falls [6], functional decline [7], institutionalisation and mortality [8], in older adult populations. Combined, these three features of mobility make up the Short Physical Performance Battery, a validated and widely applied measure of mobility in older adults [8]. Given the importance of these features in the preservation of independence and quality of life in late adulthood, mobility is here defined as the ability to walk, maintain standing balance and rise from a chair (henceforth lower-extremity functioning). Whereas all three aspects are critical components of functional mobility, there is evidence to suggest that not all domains are equally associated with cognition. For instance, in a review of longitudinal studies examining changes in mobility and cognition in older populations, gait speed was found to have a stronger correlation with a composite measure of global cognition (including tests of memory, executive functioning and processing speed) than grip strength, lower-extremity function or balance [5].

Likewise, there is reason to believe that not all domains of cognition are equally associated with mobility. First, ageing does not homogeneously disturb cognition [9]. Moreover, mobility relies more strongly on fluid aspects of cognition, such as attention, learning and sensory integration, than crystallised knowledge (e.g. language). Despite the multi-faceted nature of mobility and cognition, previous reviews have either considered multiple mobility features and a single measure of fluid cognition (henceforth referred to as cognition) [5], or a single measure of mobility and multiple cognitive features [10]. We aim to extend these findings to quantitatively analyse both the features of mobility critical for the health and quality of life in older adults and the cognitive domains implicated in ageing. By reviewing each discrete association, we can better understand the broader relationship between mobility and cognition – how far it extends and which measures are most sensitive to the underlying association. The characterisation of the mobility and cognition literature can, in turn, guide interventions targeting either domain, highlighting which measures are pertinent outcomes.

Here, we systematically review studies examining the association between objective measures of mobility and cognitive function in older adult samples. Further, we add to the literature by pooling the strength of the individual associations between these measures. We focus on common measures of mobility (gait, balance and lower-extremity functioning) and cognition (global cognitive function, memory, executive function, processing speed) affected in ageing [9]. Measures of lower-extremity function are here defined as evaluations of functional mobility assessing ability to use lower limbs to stand up from sitting. For the purpose of this review, only single-task measures of gait were included. While dual-task methodology has been widely used to assess cognitive motor interference during walking, the decline in dual-task conditions that occurs with age may be due to either cognitive or physical changes associated with ageing. Further, given the cognitive component of dual-task conditions, examining associations with cognitive tasks would lead to issues of co-linearity. Consequently, it would be unclear to ascertain whether obtained correlations were due to the shared cognitive component, or a relationship between mobility and cognition.

For each feature of physical mobility, the relation to each aspect of cognition is considered in turn. Cognitive tests are classified as executive function (including measures of working memory, selective attention, set shifting, inhibition and cognitive flexibility), memory (measures of recall, learning and recognition) or processing speed (including simple and complex reaction time measures) in accordance with a previous systematic review [11] In the context of each association, we summarise the results to date and perform *meta*-analyses of published data. Our objectives are: 1) to evaluate the evidence for associations between cognition and mobility in healthy older adults, 2) to synthesise the individual associations between aspects of mobility and cognitive domains quantitatively and 3) to explore potential sources of heterogeneity in the findings, including age, sex and differences in assessment paradigms. To the best of our knowledge, this is the first systematic review to consider how these three objective measures of mobility (gait, balance, lower-extremity function) are individually associated with memory, executive function and processing speed.

## 2 Methods

### 2.1 Data sources

We searched online for studies examining the association between physical mobility and cognitive function in healthy older adults from 1990 to February 2015 using the EMBASE and MEDLINE databases (Fig. S1). Reference lists from retrieved articles and existing reviews were manually searched for additional studies. Only English-language papers were reviewed.

### 2.2 Study selection

Two authors (ND & PE) independently reviewed the list of identified citations to assess eligibility for inclusion. Any disagreements were resolved by consensus. The following inclusion criteria were used for this review:

1. Published as a journal, article, or letter.
2. Physical mobility measured using an objective assessment of gait, balance or lower-extremity function. Self-reported measures of ability (e.g. Balance Self-Perception Test), assessments of physical activity, and of gait during dual-task conditions were excluded.
3. Cognitive ability assessed by tests of global cognition, memory, executive function or processing speed.
4. Examined an association between mobility and cognitive measures collected at the same time, a difference in mobility measures between groups that differed in cognitive function, or a difference in cognitive measures between groups that differed in mobility outcomes.
5. Included a sample of healthy adults with a mean age over 60.

### 2.3 Data extraction and analysis

The following details were extracted using a structured form: aspect of physical mobility examined (gait, balance, lower-extremity function), outcome measure of mobility feature (e.g. gait speed, score on Berg Balance test, Timed Up and Go), the cognitive domain tested (global cognition, memory, executive function and processing speed), participant demographics (sample size, mean age, sex), and results (statistically significant findings at p < 0.05, unless otherwise determined by the authors).

Studies with overlapping samples were excluded if the same aspects of mobility (e.g. gait) and cognition (e.g. executive function) were examined in both papers. In such cases, preference was given to the study with the largest sample size. For greater data homogeneity, if a study reported two levels of analysis of the same data, preference was given to the one using continuous as opposed to categorical data, as this was the more commonly used approach. Studies reporting only a composite of physical measures (e.g. gait speed + muscular weakness + fatigue) were not included. Studies that did not test for an association between mobility and cognitive measures (e.g. only used these outcomes as covariates in a model) were also not included. Moreover, measures of gait during dual-task conditions were not included (for review see [12]).

To facilitate comparability, the directions of associations were reversed if lower scores indicated better performance. For example, associations using walking time and the Trail Making Test (e.g. [4], [13]), were reversed to match the direction of associations using gait speed and verbal fluency.

When multiple measures of the same construct were included in one study, we first selected the measures most commonly used to maximise comparability between studies. This led to the selection of gait speed whenever possible, and the construct that most closely resembled it when not (i.e. walking time and pace). Similarly, regarding studies of memory, measures of immediate recall were preferred to those of delayed recall due to cross-study variation in the levels of interference during the delay-period.

If a study contained more than one assessment of a cognitive measure, and the multiple measures were deemed comparable, a study-level pooled effect size was calculated across measures of the same construct (i.e. the Stroop and Trail Making Test in [14]).

It is important to note that there is always some overlap between the physical mobility areas of speed, balance and lower-extremity function measures. In order to separately consider the relationships between each mobility feature and cognition, we split mobility tasks in accordance with their focus on propulsion, balance or power, respectively. For example, although gait measures were reviewed in the gait section, if the measure had balance as a primary focus (e.g. mediolateral body sway in [14]), we reported this finding within the balance section even if it was measured during gait.

When possible, results are presented after controlling for age, sex, and education, but before adjusting for additional factors (e.g. disease, medication, social class).

All included tests were chosen prior to extraction of results.

### 2.4 Data synthesis

The meta-analyses were conducted using Comprehensive Meta-Analysis software, version 2 (Biostat Inc., NJ, USA). Effect sizes were measured using standardised mean differences and are reported alongside 95% confidence intervals. In light of expected differences in study sample and design, random-effects models were used to calculate the pooled mean effect size. Heterogeneity across studies was tested using Q-statistics [15]. The I^2^ index [15] was additionally used to assess consistency between studies, as it does not inherently depend on the number of studies in the meta-analysis. As suggested by Higgins et al. (2003), the I^2^ index was interpreted to represent low, moderate or high inconsistency, if equal to I^2^ values of 25%, 50% and 75%, respectively [16]. To address the possibility of publication bias, we examined funnel plots [17] and used Begg and Mazumdar rank correlations [18]. As a minimum of 3 studies is required to compute Begg and Mazumdar rank correlations, this analysis was not possible in all cases. The Trim and Fill procedure [19] was applied if evidence of publication bias was noted. When only confidence intervals were given, *p*-values were calculated as described by Altman and Bland [20]. If a study did not report the direction of an association, authors were contacted. If further information was not obtained, results were outlined in review tables but not included in the meta-analyses.

## 3 Results

### 3.1 Study selection

Titles and abstracts of all identified articles (n = 642) were screened. After full-text review, 26 articles met the stipulated eligibility criteria (Fig. S2). Overall, a total of 26,355 participants were included.

### 3.2 Gait

A total of 25 studies examined the relationship between gait and cognition, outnumbering the amount of studies using balance (N = 5) or lower-extremity function (N = 6) as an outcome measure of mobility (Table 1). Most commonly, the outcome measure was self-paced gait speed (72%), obtained using electronic walkways (e.g. GaitMat in [21]) or by calculating time to complete a given distance (e.g. Soumare et al. [22]).

**Fig. 1:**
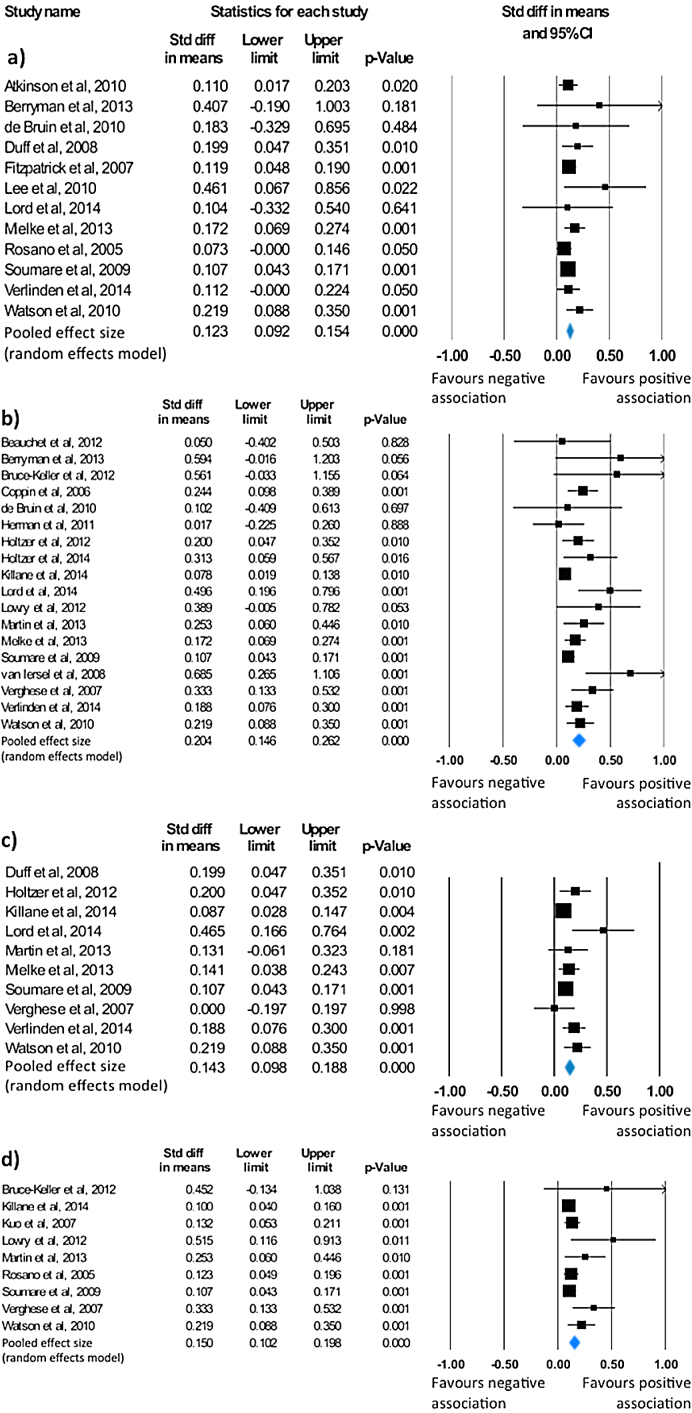
Statistical summary and forest plot of effect sizes for the association between a) gait and global cognition, b) gait and executive function, c) gait and memory and d) gait and processing speed.

Fifteen of the included studies examined the association between gait and global cognition in healthy older adults [4], [22], [23], [24], [25], [26], [27], [28], [29], [30], [31], [32], [33], [34], [35]. The most common measure of global cognition, employed in 80% of studies, was the Mini-Mental State Examination (MMSE) or its modified version, the 3MS. The majority of studies (n = 9) observed that slower gait speed was associated with worse global cognition [4], [22], [23], [24], [25], [26], [27], [28], [29]. A meta-analysis of 12 studies revealed a small effect size of 0.12 (95% CI = 0.09 to 0.15, *p* < 0.001; Fig. 1A) in favour of a positive association between gait and global cognition, suggesting that older adults with faster gait performed better on measures of global cognition. Studies were not significantly heterogeneous (Q = 9.82, *p* = 0.547, I^2^ = 0). However, as revealed by the asymmetrical funnel plot (Fig. S3), and supported by Begg and Mazumdar’s rank correlation (τ = 0.45, *p* = 0.04), there was significant indication of publication bias. Accordingly, the Trim and Fill procedure was applied to impute missing studies, resulting in a mean effect size of 0.11 (95% CI = 0.08 to 0.14).

A total of 19 studies addressed the association between measures of gait and executive functioning [13], [14], [21], [28], [29], [30], [31], [32], [33], [34], [35], [36], [37], [38], [39], [40], [41]. Significant findings were reported in 13 studies [13], [14], [21], [22], [26], [29], [30], [35], [36], [37], [38], [40], [41], all of which suggested that older adults with faster gait performed better on tests of executive function (Table 1). A meta-analysis of 18 published results found an overall mean effect size of 0.2 (95% CI = 0.15 to 0.26, *p* < 0.001; Fig. 1B), indicating a moderate association between gait and executive function measures. Moderate heterogeneity was found across studies (Q = 34.81, p = 0.007, I^2^ = 51.17). To explore this heterogeneity, and in light of the high variability in measures of executive functioning, post-hoc subgroup analyses were performed (Fig. S4). Subgroup analysis demonstrated more prominent effects for studies using the Stroop test, a combination of executive function tasks and the Digit Span test. Given indication of publication bias (τ = 0.41, *p* = 0.02; Fig. S5), the Trim and Fill procedure was applied, yielding a mean effect size of 0.17 (95% CI = 0.11 to 0.24).

Our search identified 11 studies that examined the relationship between measures of gait and memory [4], [22], [26], [28], [29], [33], [35], [37], [39], [40], [41]. Eight studies reported significant findings, and all significant findings pointed towards a positive association between these two domains [4], [22], [28], [29], [36], [35], [37], [39]. A meta-analysis of 10 studies assessing gait and memory showed an overall small mean effect size of 0.14 (95% CI = 0.1 to 0.19; *p* < 0.001; Fig. 1C), representing a small association between greater gait speed and performance on memory tests. There was no significant heterogeneity across studies (Q = 13.38, *p* = 0.15), with only a low level of inconsistency (I^2^ = 32.73). There was also no indication of publication bias (τ = 0.27, p = 0.14; Fig. S6).

Nine of the identified studies examined the relationship between gait and processing speed [21], [22], [27], [29], [31], [39], [40], [41], [42], eight of which observed a positive association between the two domains [21], [22], [27], [29], [39], [40], [41], [42]. While the Digit symbol test was the most common measure of processing speed, others also used part A of the Trail Making test [22], the Boxes and Digit copying tests [29], and a choice reaction time test [39]. A meta-analysis of the 9 identified studies resulted in a small mean effect size of 0.15 (95% CI = 0.1 to 0.2; *p* < 0.001; Fig. 1D) in favour of a positive association between gait speed and performance on processing speed tasks. Due to indication of publication bias (τ = 0.64, p = 0.01; Fig. S7), the Trim and Fill procedure was applied, adjusting the mean effect size to 0.14 (95% CI = 0.08 to 0.19). No significant heterogeneity (Q = 13.51, *p* = 0.1, I^2^ = 40.79) was observed.

### 3.3 Lower-extremity function

A total of six studies addressed the relationship between lower-extremity function and cognition (Table 2). Half of the identified studies used the Timed Up and Go (TUG) test to assess lower-extremity function [30], [34], [43], while the other half used the Chair Stand test [23], [25], [27].

**Fig. 2:**
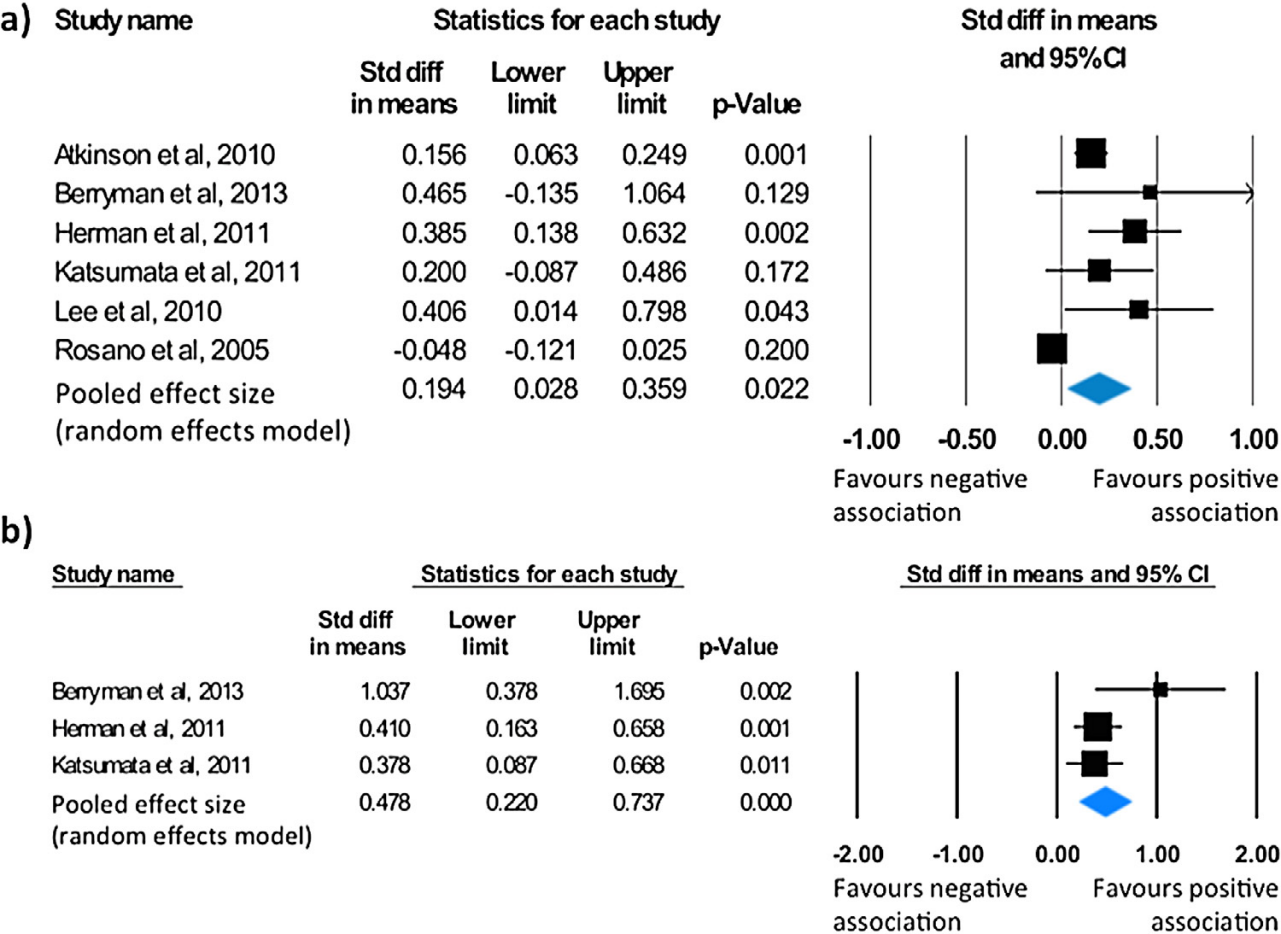
Statistical summary and forest plot of effect sizes for the association between a) lower-extremity function and global cognition, and b) lower-extremity function and executive function.

Six studies examined the association between lower-extremity function and global cognition [23], [25], [27], [30], [34], [43]. All included studies either used the Mini-Mental State Examination or its modified version, the 3MS, as a measure of global cognition. Lower-extremity function was assessed with the Timed Up and Go test and the Chair Stand test. A meta-analysis of all six studies showed a small mean effect size of 0.19 (95% CI = 0.03 to 0.36, *p* = 0.022; Fig. 2A). Heterogeneity (Q = 24.75, *p* < 0.001), with a high level of inconsistency (I^2^ = 79.8), was observed between studies. There was no indication of publication bias (τ = 0.07, p = 0.85; Fig. S8).

Three studies addressed the association between lower-extremity function and executive function [30], [34], [43]. In all three cases, the Timed Up and Go test was used to measure lower-extremity function. All studies reported a significant link between executive functioning and performance on the TUG (Table 2). A meta-analysis of the published results revealed a moderate mean effect size of 0.48 (95% CI = 0.22 to 0.74, *p* < 0.001; Fig. 2B) in favour of a positive association between measures of lower-extremity function and executive function. Studies were not significantly heterogeneous (Q = 2.79, *p* = 0.25) although a low level of inconsistency was noted (I^2^ = 28.3). The Begg and Mazumdar rank correlation (τ = 0.33, *p* = 0.6) and the symmetrical funnel plot (Fig. S9) suggest publication bias was absent.

Only one study examined the association between measures of lower-extremity function and memory. Katsumata and colleagues (2011) found that participants that were faster to complete the Timed Up and Go test also performed better on a test of visual memory [43].

Similarly, the only study to look at the relationship between lower-extremity function and processing speed reported a positive association between performances on the Chair stands test and the Digit symbol substitution test [27].

### 3.4 Balance

A total of five studies examined the relationship between balance and cognition (Table 3). A variety of tests were used as indicators of balance, including standardised tests (Berg Balance Test in [34], and Standing Balance Test in [27]) and measures obtained from quantitative gait analysis (mediolateral body sway in [14]). The remaining measures focused on tandem walking [28] and tandem stance time [25].

**Fig. 3:**
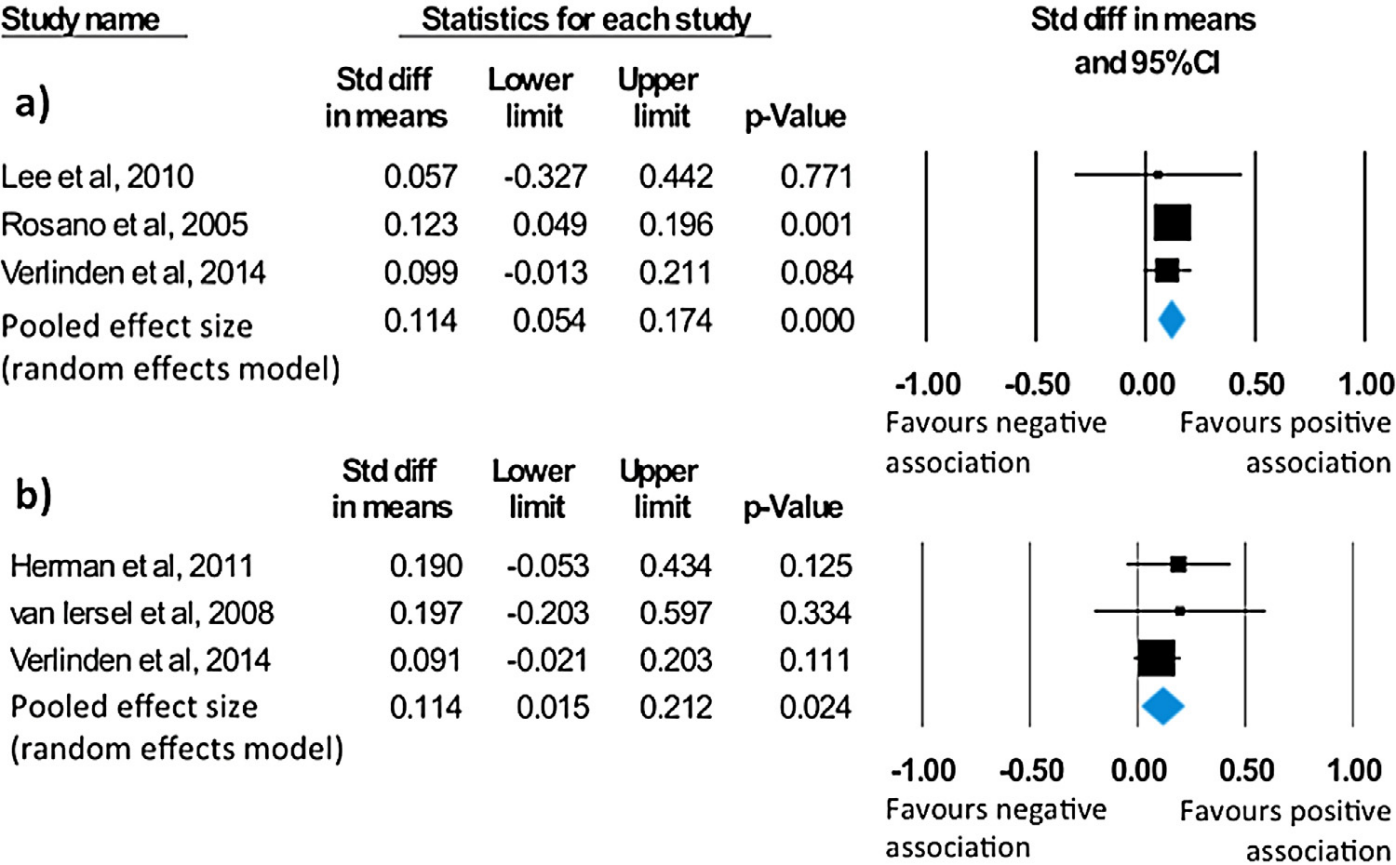
Statistical summary and forest plot of effect sizes for the association between a) balance and global cognition, and b) balance and executive function.

Four studies conducted analysis on the relationship between balance and global cognition [25], [27], [28], [34]. Cognition was assessed with mental state examinations (MMSE or 3MS) in all cases. Reported findings were not significant for three studies [25], [28], [34]. However, the largest study [27], found that better performance on the Standing Balance Test was associated with increased global cognitive status, as indicated by the 3MS. A meta-analysis conducted on the 3 studies reporting directionality showed a significant, albeit small, effect size of 0.11 (95% CI = 0.05 to 0.17, *p* < 0.001; Fig. 3A). Across studies, no heterogeneity (Q = 0.21, *p* = 0.9, I^2^ = 0) or indication of publication bias (τ = 0.3, *p* = 0.6; Fig. S10) was observed.

As for executive function, a total of three studies reported analysis on the relationship between balance and executive function [14], [28], [34]. All studies used a combination of the following standard tests of executive functioning: digit span, verbal fluency, the Trail Making Test (TMT) and the Stroop test. Although van Iersel et al. (2008) reported a significant association between performance on the Stroop test and mediolateral angular velocity, an index of balance, the overall association between all tests of executive function and indices of balance used in their study was not significant [14]. The remaining studies did not report any significant results [28], [34]. Based on these three studies, the meta-analysis of balance-executive function associations revealed a significant mean effect size of 0.11 (95% CI = 0.02 to 0.21, *p* = 0.02; Fig. 3B) in favour of a positive association between the two measures. No heterogeneity was observed across studies (Q = 0.75, *p* = 0.69, I^2^ = 0). Moreover, there was no indication of publication bias (τ = 0.33, *p* = 0.6; Fig. S11).

Two of the identified studies examined the association between measures of balance and memory [14], [28]. Memory was assessed with a verbal learning test [28], and a combination of episodic and visual recognition memory tests [14]. No significant findings were reported.

Finally, a single study reported on the relationship between measures of balance and processing speed in older adults. Rosano and colleagues (2005) found that participants who performed better on the Standing balance test also performed faster on the Digit symbol substitution test [27].

## 4 Discussion

We systematically reviewed cross-sectional reports of relationships between features of mobility and subdomains of cognition. This review had three aims: 1) to evaluate the evidence for associations between cognition and mobility in healthy older adults, 2) to pool the individual associations between aspects of mobility and cognitive domains quantitatively and 3) to explore potential sources of heterogeneity in the findings, including age, sex and measurement type.

With regard to aim 1, the reviewed evidence suggests that individuals with better mobility perform better on assessments of global cognition, executive function, memory and processing speed. While reports of non-significant findings were also identified, the direction of all significant associations was unanimously positive, thus further encouraging our conclusion.

With regard to aim 2, we conducted meta-analyses to pool results from individual associations between features of mobility and cognitive domains (Table 4). Wherever sufficient studies were available for analysis, significant, albeit mostly small, effect sizes were obtained.

In terms of gait, a recent systematic review by Morris and colleagues (2016) found evidence for associations with measures of global cognition, executive function, visuospatial cognition and language [10]. Here, we extend these findings by highlighting a significant association with memory and processing speed, and providing quantitative evidence in support of the reviewed relationships.

Similar to gait findings, lower-extremity function was associated with global cognition and executive function. While the association with executive function yielded the largest mean effect size (0.48), this must be interpreted with caution given the small number of studies in this analysis. Only one study examined lower-extremity function measures in relation to either memory [43] or processing speed [27], yet both reported significant findings.

Balance measures were also significant overall, however few studies examined the relationship between balance and cognition. As was the case with lower-extremity function, significant mean effect sizes were obtained for the associations with global cognition and executive function, but there were insufficient studies to conduct meta-analyses for memory and processing speed.

The pattern observed in the results from our meta-analyses was partially reflected in the few studies that examined all three mobility features. While two studies found that balance was not associated with cognition despite associations with gait or lower-extremity of function [25], [34], Rosano and colleagues (2005) found significant associations across all mobility features [27]. As the latter was a much larger study, it may be the case that the former studies lacked the power to identify a balance-cognition relationship. Accordingly, despite caution, our overall finding that all measures of mobility were associated with cognition is in line with the largest individual study to assess multiple measures of mobility. Therefore, while the mobility literature often focuses on gait measures, our findings suggest that alternative measures, such as tests of balance and lower-extremity of function, may also serve as valuable mobility outcomes in interventions targeting either domain.

As for cognitive-specificity, only the gait literature offered sufficient studies to conduct meta-analyses with each cognitive domain. For gait, effect sizes were found to be significant and consistent across cognitive domains (0.1-0.19). This consistency in findings suggests that the association between gait and cognition is not exclusive to one cognitive domain. A similar pattern was observed by individual gait studies that measured at least 3 cognitive domains. Of these, 3 found significant correlations in two of the three domains [35], [39], [41], while four studies reported significant correlations across all cognitive measures reviewed here [22], [26], [28], [29].

Overall, our findings argue in favour of a global association between mobility and cognitive measures, although more, well-powered, research is warranted to ascertain the relationship between balance and cognition. The broader conclusions we may draw from this, their limitations, and the nature of these relationships will be addressed next. Finally, in reference to our third aim, we will explore the role of sex, age and assessment type in the reviewed associations.

### 4.1 The nature of the relationship between cognition and mobility

There are a number of interpretations of the observed positive associations between cognition and mobility in older adults. As with any cross-sectional association, it is not possible to determine the direction of causality of the reported relationship. Longitudinal findings or intervention studies may shed light on the direction of causality between cognition and mobility.

Age-related changes in cognition may be driving changes in the mobility of older adults. Firstly, physical mobility relies on cognitive processes to anticipate and adapt to the moving environment while maintaining postural control and motor coordination [27], [44]. Gait, for instance, requires the interplay of attention, executive function, and visuospatial processing. Moreover, gait also requires monitoring of motor functions from the motor cortex, basal ganglia and cerebellum. Thus, a decrease in cognitive function may have detrimental effects on mobility functioning. The interdependence between mobility and cognition may become even stronger with age, as increased cognitive monitoring is required to compensate for age-related declines in the sensorimotor system [45]. Consistent with this line of reasoning, a longitudinal study of older adults found that cognitive decline preceded mobility impairments [46].

Conversely, reduced mobility may aggravate cognitive decline. Decreased mobility can limit social interactions, engagement in leisure activities and increase risk of depression − all of which could, in turn, have detrimental effects on cognitive function [47], [48], [49]. Accordingly, there is evidence to suggest that subjects with mobility impairments at baseline had a significantly greater risk of developing cognitive disabilities [26], [41]. However, the cross-sectional nature of this review makes it impossible to disentangle the directionality of the mobility-cognition relationship.

It is also possible that mobility and cognition are affected by a “common cause”, in which some common factor, such as general degeneration of the central nervous system, is responsible for a decline in both functions. This theory has been proposed for the relationship between sensory changes and cognition [50], and could arguably also apply to the association between mobility and cognition. A common cause would, however, suggest that all aspects of mobility and cognition are equally associated. Our findings, with balance showing a weaker link to cognition than other mobility measures, do not support this. Moreover, the variance in magnitudes of effect sizes across cognitive domains suggests that the modularity of cognition may also be observed in the strength of its relationship with mobility.

### 4.2 Methodological considerations

Studies varied in terms of inclusion criteria, experimental design and, perhaps most crucially, assessment paradigms.

In terms of cognitive measures used, two concerns must be addressed. First, the majority of studies used the MMSE or its modified version, the 3MS, to assess global cognition. The MMSE and 3MS were designed as screening tools for cognitive impairments. Consequently, when acting as measures of global cognitive function, these measures are prone to ceiling effects and show very little variance in cognitively healthy samples [51]. It is also important to note that these cognitive screens are heavily weighted towards language and memory function, largely neglecting other cognitive domains, such as processing speed. Few studies used a summary score of a breadth of cognitive tests as a measure of global cognition [4], [26]. A composite score that includes a range of cognitive domains might be more representative of global cognitive function than cognitive screening tests like the MMSE, and thus more informative for future studies.

Second, studies varied in the paradigms used to measure memory and executive function. As revealed by a post-hoc subgroup analysis, this was a likely cause for the heterogeneity observed across studies analysing the association between executive function and gait. In light of the diverse nature of executive function, this is perhaps unsurprising. Nonetheless, interpretation of heterogeneity depends on whether effects show the same direction, or not [52]. Given the positive direction of all associations between gait and measures of executive function, it is arguable that the identified heterogeneity does not undermine the results of this meta-analysis.

It should be noted that cognition also comprises visuospatial processing, an aspect of cognition that also declines with age [9], and may impact gait control [4]. Unfortunately, the classification of cognitive domains is often an impure task. Measures of memory (e.g. Spatial memory recognition task from CANTAB in [35]), executive function (e.g. the Trail Making Test in [22]) and processing speed (e.g. the Digit Symbol Substitution test in [21]) also involve visuospatial components. Consequently, disentangling measures of visuospatial processing from other cognitive domains would be somewhat arbitrary. We did not, therefore, include it as a separate cognitive domain in our review.

As for measures of mobility, significantly fewer studies examined balance or lower-extremity of function, than gait. Within each aspect of mobility, comparability was facilitated by the overlap observed in assessments used. Our focus on gait speed stemmed from a concern for data homogeneity. To date, gait speed is the most common gait parameter in the mobility and cognition literature. However, gait speed is a global marker of gait disturbance related to central, but also peripheral, neuromuscular dysfunction and other gait parameters (e.g. step time, stride length and stride time variability) have emerged as more specific correlates of cognitive measures [40], [41]. Whereas several of the studies included here also reported alternative gait measures (e.g. stride time variability in [14], step length variability in [35]), it was beyond the scope of this review to evaluate how multiple gait parameters relate to individual cognitive domains. Nonetheless, focusing on one measure of gait (i.e. speed) is a limitation of this review. Further, clinical measures of mobility are often performed in controlled environments that require less mental processing and relationships may be stronger between cognitive tests and mobility measures performed in community settings.

Regarding participant characteristics, studies varied greatly in terms of sex (range 36–100% female) and mean age (range 62–80 years). It has been suggested that the cognitive benefit of physical activity may be greater in women than men [53], but the effect of sex on the relationship between mobility and cognition is not yet clear. Our meta-regressions with “%-female in study” as independent variable were not significant, although the small number of studies in these analyses limits the power of such meta-regressions (*p*-values ranged from 0.14 to 0.98; Supplementary materials). Similarly, our meta-regressions with age did not reveal any significant associations between effect size and mean age of participants (*p*-values ranged from 0.26 to 0.77; Supplementary materials). Nevertheless, further research examining the effect of age and sex on the relationship between mobility and cognition is necessary.

Finally, only published work was included in this review. While this may have raised susceptibility to publication bias, restricting the search to published results serves as a guarantee of peer-reviewed quality in included reports.

### 4.3 Conclusion

In conclusion, this systematic review suggests a positive association between mobility and cognitive function in healthy older adults. Interestingly, studies examining the link between cognition and balance, although sparse, suggest that this aspect of mobility is less likely to show a significant association with cognitive measures. Building on from our results, future studies should aim to disentangle the directionality of the relationship between cognition and mobility. Further research into the nature of this association may lead to the identification of candidates for early detection of age-related impairments.

## Conflicts of interest

None.

## Figures and Tables

**Table 1:** Characteristics of studies on the relationship between gait and cognition.

First Author, Ref.	N	Mean Age	% Female	Gait Measure	Cognitive Measure	Relationship
Atkinson [23]	1793	70.3 ± 3.7	100	Gait speed (usual pace, 6 m)	3MS	↑
Beauchet [36]	78	69.8 ± 0.8	59	Stride time variability (SMTEC system, 10 m walkway)	Digit span	↑
					TMT	Not significant
					Stroop	Not significant
Berryman [30]	48	70.5 ± 5.3	58	Fast vs. Slow walkers (usual pace, 10 m)	MMSE	Not significant
					Stroop	↑
Bruce-Keller [31]	50	74.2 ± 7.8	42	Gait speed (GAITRite system)	MMSE	Not significant
					Verbal fluency	Not significant
					Digit symbol	Not significant
Coppin [13]	737	72.7 ± 5.9	54	Gait speed (usual pace, 7 m)	TMT	↑
De Bruin [32]	62	72.5 ± 5.9	45	Gait speed (GAITRite system)	MMSE	Not significant
					Inhibition	Not significant
Duff [4]	675	73.2 ± 5.8	57	Walking time (usual pace, 15.24 m)	RBANS	↑
					Immediate memory (RBANS)	↑
Fitzpatrick [24]	3070	78.6 ± 3.3	46	Gait speed (usual pace, 15 feet)	3MS	↑
Hausdorff [33]	43	71.9 ± 6.4	51	Gait speed (distance at usual pace for 2 min)	MMSE	Not significant
					Stroop	Not significant
					10-word-pairs verbal learning test	Not significant
Herman [34]	265	76.4 ± 4.3	58	Dynamic gait index	MMSE	Not significant
					Digit span	Not significant
					Verbal fluency	Not significant
Holtzer [37]	671	79 ± 5.2	60	Gait speed (GAITRite system)	(Executive function) Composite	↑
					Free recall (FCSRT)	↑
Holtzer [38]	247	76.5 ± 7.2	55	Gait speed (GAITRite system)	Flanker task	↑
Killane [39]	4344	62 ± 8	55	Gait speed (GAITRite system)	Color trail test	Not significant
					Verbal fluency	Not significant
					10-word verbal learning test	↑
					Choice RT	↑
Kuo [42]	2481	71 ± 7.7	51	Gait speed (usual pace, 6.1 m)	Digit symbol	↑
Lee [25]	107	73.8	100	Gait speed (usual pace, 6 m)	MMSE	↑
Lord [35]	184	69.4 ± 7.7	58	Pace (GAITRite system)	MoCA	Not significant
					(Executive function) Composite	↑
					Spatial recognition memory, Pattern recognition memory and Paired associates learning (CANTAB)	↑
Lowry [21]	106	77 ± 5.8	70	Gait speed (usual pace, GaitMat II)	TMT	↑
					Digit symbol	↑
Martin [40]	422	72 ± 7	44	Gait speed (GAITRite system)	(Executive function) Composite	↑
					Hopkins verbal learning test and Delayed figure reproduction (RCF)	Not significant
					Digit symbol	↑
Mielke [26]	1478	78.8 ± 4.1	52	Gait speed (usual pace, 7.65 m)	(Gobal) Composite	↑
					TMT/Verbal fluency	↑
					Logical memory and Auditory verbal learning (WMS-R)	↑
Rosano [27]	2893	73.6 ± 2.9	52	Gait speed (usual pace, 6 m)	3MS	↑
					Digit symbol	↑
Soumare [22]	3769	73.5 ± 4.7	62	Maximum gait speed (6 m)	MMSE	↑
					TMT	↑
					Benton visual retention test	↑
					TMT A	↑
Van Iersel [14]	100	80.6 ± 4	36	Gait speed (GAITRite system)	Stroop	↑
					TMT	Not significant
Verghese [41]	399	79.2 ± 4.9	56	Pace (GAITRite system)	Verbal fluency	↑
					Digit span	↑
					Free and cued selective reminding test	Not significant
					Digit symbol	↑
Verlinden [28]	1232	66.3 ± 11.8	55	Pace (GAITRite system)	MMSE	↑
					Stroop/Verbal fluency	↑
					15-word verbal learning test	↑
Watson [29]	909	75.2 ± 2.8	51	Gait speed (usual pace, 20 m)	3MS	↑
					Executive interview	↑
					The Buschke selective reminding test	↑
					The Boxes and Digit copying tests	↑

Abbreviations: 3MS, Modified Mini-Mental State Examination; MMSE, Mini-Mental State Examination; MoCA, Montreal Cognitive Assessment; RBANS, Repeatable Battery for the Assessment of Neuropsychological Status; TMT, Trail Making Test; TMT A, Trail Making Test part A; FCSRT, Free and Cued Selective Reminding Test; RCF, Rey Complex Figure; WMS-R, Wechsler Memory Scale-Revised; Choice RT, Choice reaction time.

**Table 2:** Characteristics of studies on the relationship between measures of lower-extremity function and cognition.

First Author, Ref.	N	Mean Age	% Female	LEF Measure	Cognitive Measure	Relationship
Atkinson [23]	1.793	70.3 ± 3.7	100	Chair stands	3MS	↑
Berryman [30]	48	70.5 ± 5.3	58	Timed Up and Go	MMSE	Not significant
					Stroop	↑
Herman [34]	265	76.4 ± 4.3	58	Timed Up and Go	MMSE	↑
					Digit span	↑
					Verbal fluency	↑
Katsumata [43]	192	85.1 ± 3.2	73	Fast/normal vs. Slow (TUG)	J-MMSE	Not significant
					Verbal fluency	Fast/normal > Slow (TUG)
					Scenery Picture Memory test	Fast/normal > Slow (TUG)
Lee [25]	107	73.8	100	Chair stands	MMSE	↑
Rosano [27]	2893	73.6 ± 2.9	52	Chair stands	3MS	Not significant
					Digit symbol	↑

Abbreviations: LEF, Lower-extremity function; TUG, Timed Up and Go; MMSE, Mini-Mental State Examination; 3MS, Modified Mini-Mental State Examination; J-MMSE, Japanese Mini-Mental State Examination.

**Table 3:** Characteristics of studies on the relationship between measures of balance and cognition.

First Author, Ref.	N	Mean Age	% Female	Balance Measure	Cognitive Measure	Relationship
Herman [34]	265	76.4 ± 4.3	58	Berg Balance Test	MMSE	Not significant
					Digit span	Not significant
					Verbal fluency	Not significant
Lee [25]	107	73.8	100	Tandem stance (time)	MMSE	Not significant
Rosano [27]	2893	73.6 ± 2.9	52	Standing Balance Test	3MS	↑
					Digit symbol	↑
Van Iersel [14]	100	80.6 ± 4	36	ML displacement ML angular velocity	TMT	Not significant
					Stroop	Not significant
					Paired Associates Learning/Pattern Recognition Memory	Not significant
					TMT	Not significant
					Stroop	Not significant
					Paired Associates Learning/Pattern Recognition Memory	↑
Verlinden [28]	1232	66.3 ± 11.8	55	Tandem walk	MMSE	Not significant
					Stroop/Verbal fluency	Not significant
					Verbal recall	Not significant

Abbreviations: ML displacement, Mediolateral displacement; ML angular velocity, Mediolateral angular velocity; TMT, Trail Making Test; MMSE, Mini-Mental State Examination; 3MS, Modified Mini-Mental State Examination.

**Table 4:** Summary of mean effect sizes obtained for each reviewed association.

		Cognition			
		Global Cognition	Executive Function	Memory	Processing Speed
Mobility	Gait	0.11** (N = 12)	0.17** (N = 18)	0.14** (N = 10)	0.14** (N = 9)
	Lower-extremity function	0.19^§^ (N = 6)	0.48** (N = 3)	N/A (N = 1)	N/A (N = 1)
	Balance	0.11** (N = 3)	0.11^§^ (N = 3)	N/A (N = 2)	N/A (N = 1)

N/A: Not available because mean effect sizes were only calculated when more than 3 studies were identified.

^§^*p* < 0.05; *p <0.01; **p < 0.001.
