# Supplementary material for: A systematic review and meta-analysis of cross-sectional studies examining the relationship between mobility and cognition in healthy older adults
Source: Gait Posture. 2016 Oct;50:164–74. doi: 10.1016/j.gaitpost.2016.08.028 (PMC5081060; doi:10.1016/j.gaitpost.2016.08.028)
Supplement: Supplementary file 1 [file mmc1.docx]

**SUPPLEMENTARY MATERIAL**

1. exp gait/
2. Physical mobility.mp
3. Physical performance.mp
4. 1 or 2 or 3
5. exp cognition/
6. exp memory/
7. 5 or 6
8. 4 an 7
9. limit 8 to “all aged 65 or over”
10. limit 9 to (journal article or letter)

Figure S1. Search criteria used for MEDLINE


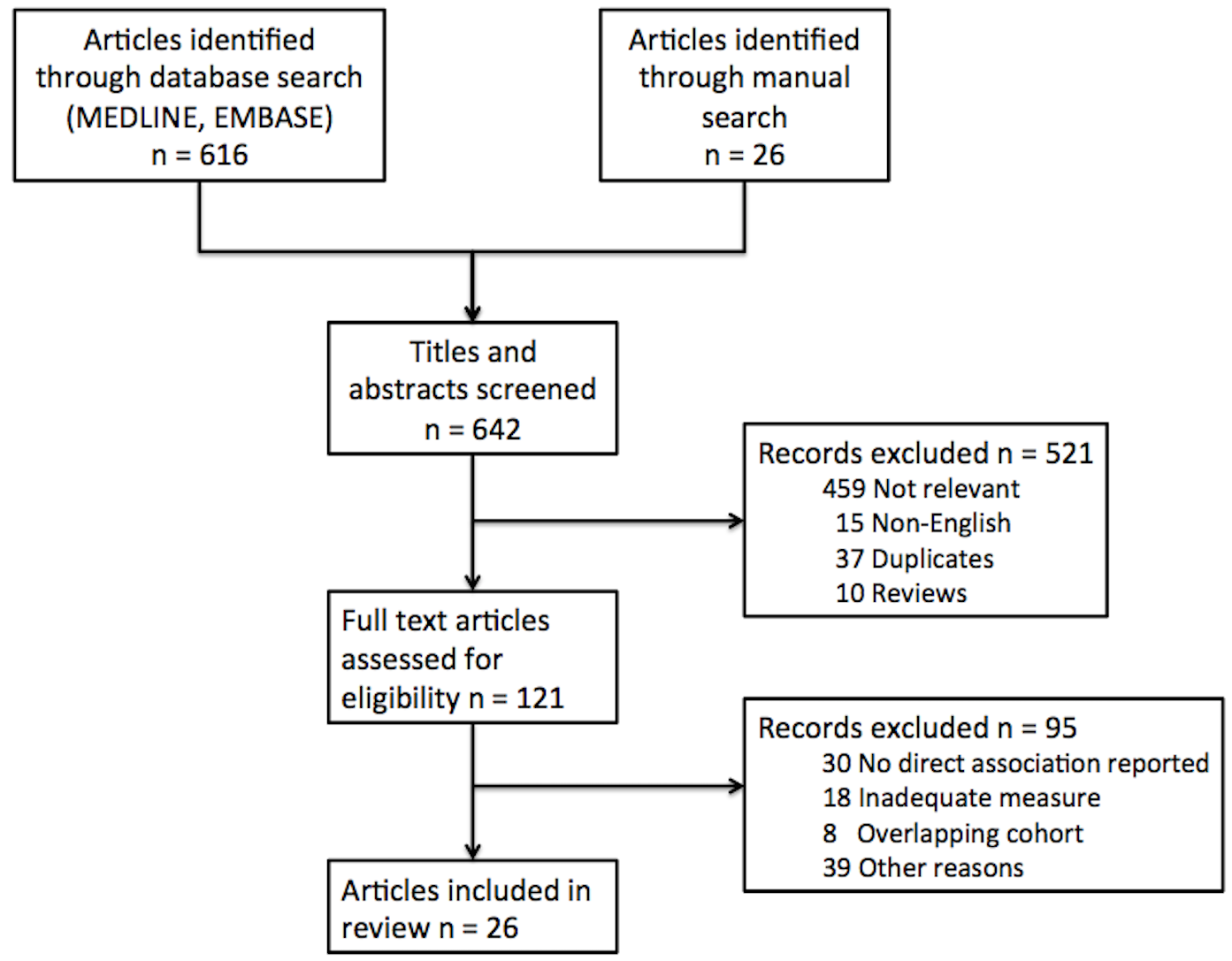


Figure S2. Identification and attrition of studies.


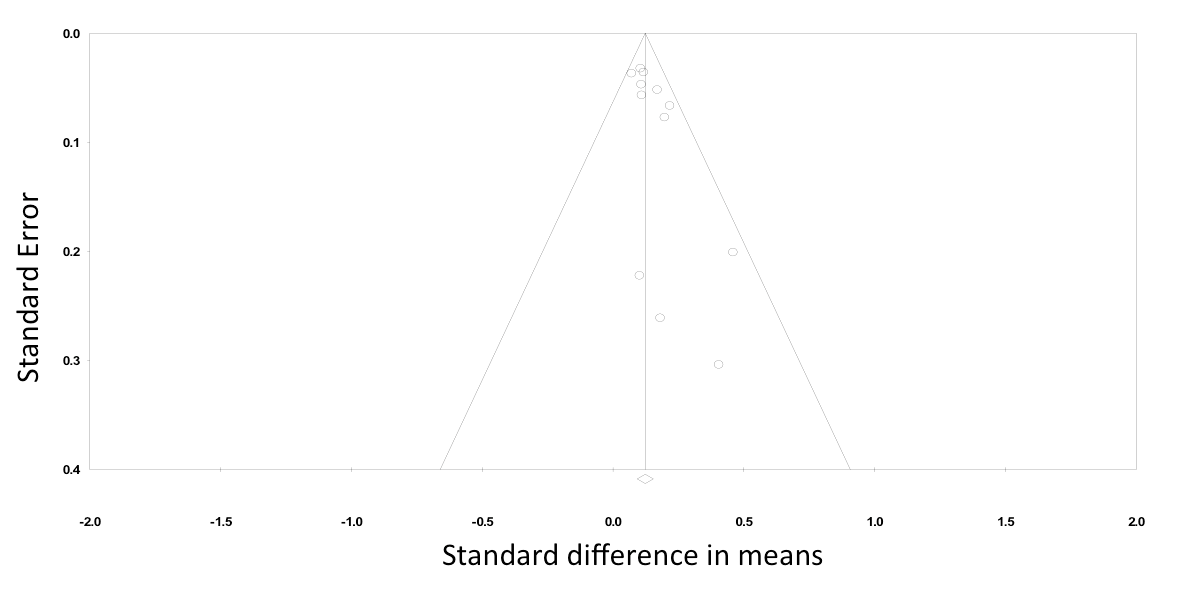


Figure S3. Gait and global cognition: Funnel plot of standard errors plotted against study effect sizes.


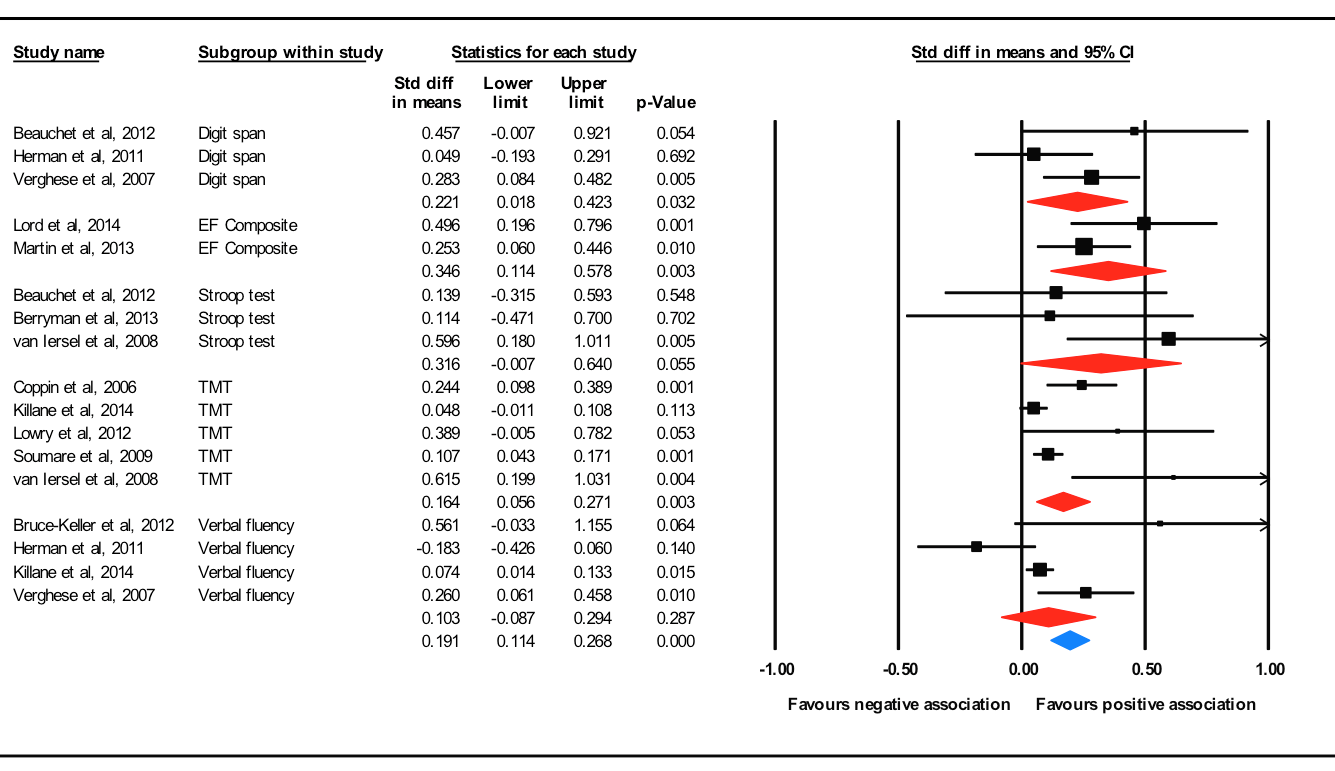


Figure S4. Statistical summary and forest plot of effect sizes for the subgroup analysis of the gait-executive function association, with five clusters based on task type. Only tasks used by more than one study were included in this analysis.

It is worth noting that this post-hoc analysis is merely exploratory, as it allowed the inclusion of the same cohort twice in the cases when multiple tests were used in one study, to allow comparison between effects for each test. Abbreviations: TMT, Trail Making Test; EF, Executive Function.


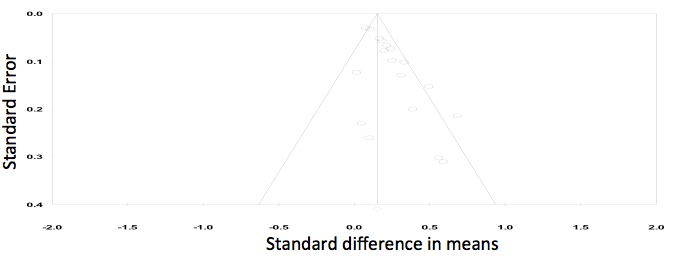


Figure S5. Gait and executive function: Funnel plot of standard errors plotted against study effect sizes.


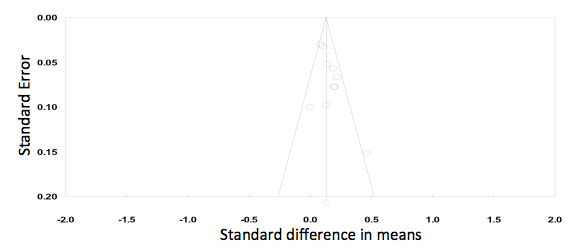


Figure S6. Gait and memory: Funnel plot of standard errors plotted against study effect sizes.

**
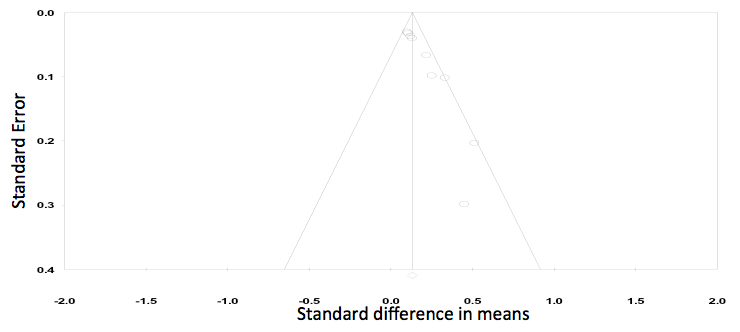
**

Figure S7. Gait and processing speed: Funnel plot of standard errors plotted against study effect sizes.


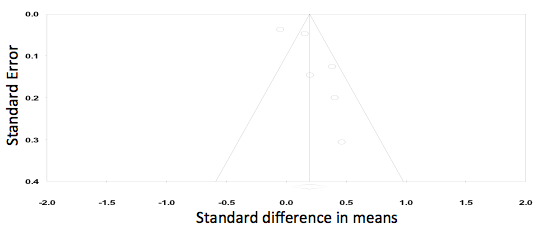


Figure S8. Lower-extremity function and global cognition: Funnel plot of standard errors plotted against study effect sizes.


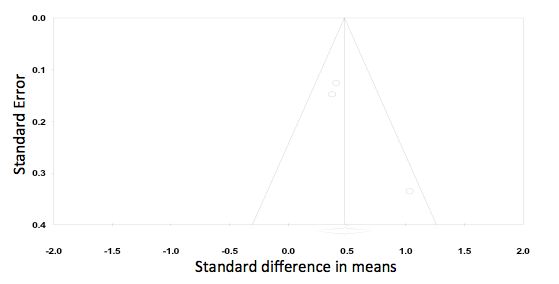


Figure S9. Lower-extremity function and executive function: funnel plot of standard errors plotted against study effect sizes.


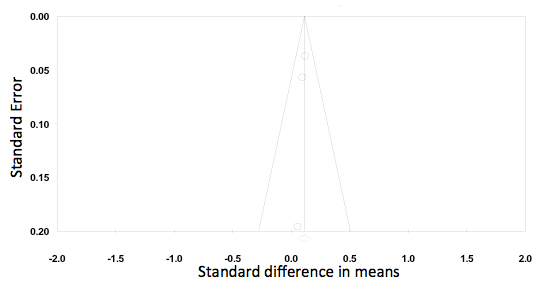


Figure S10. Balance and global cognition: funnel plot of standard errors plotted against study effect sizes.


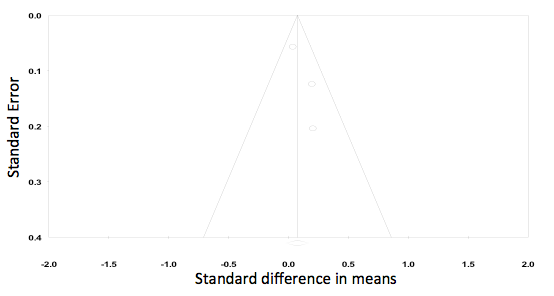


Figure S11. Balance and executive function: funnel plot of standard errors plotted against study effect sizes.


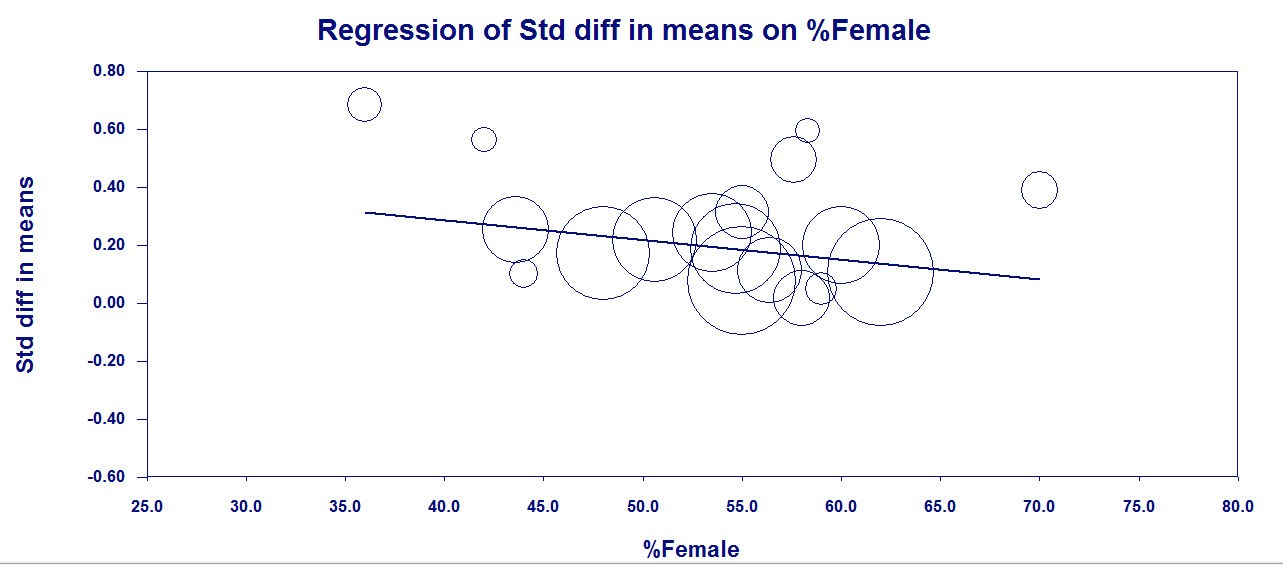


Figure S12. Gait and executive function - regression of standard difference in means on % female (random-effects model).


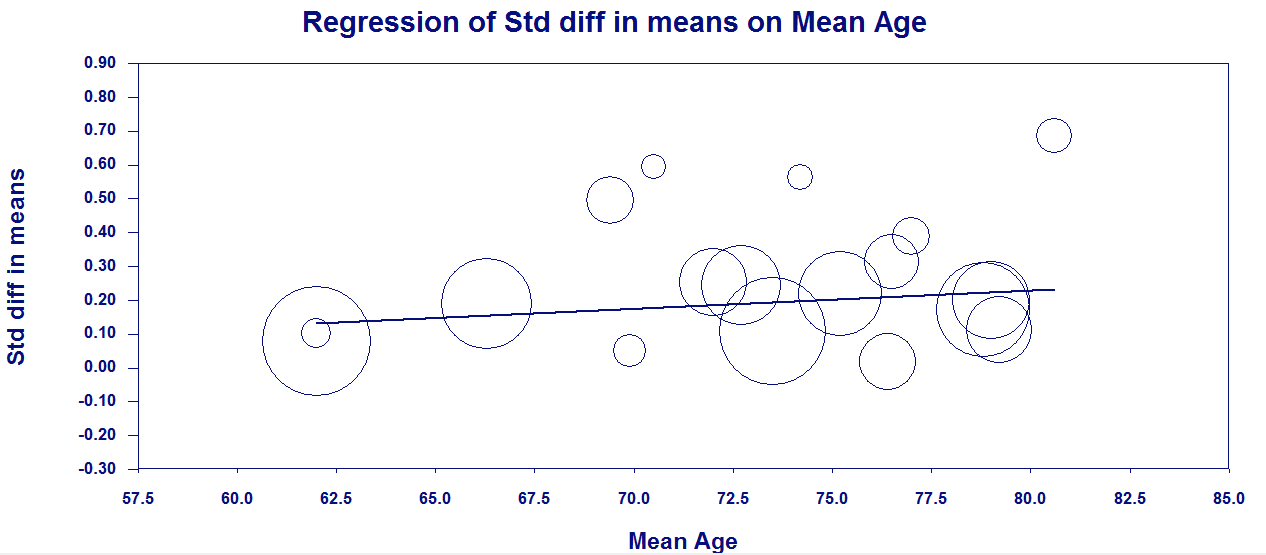


Figure S13. Gait and executive function - regression of standard difference in means on mean age (random-effects model).


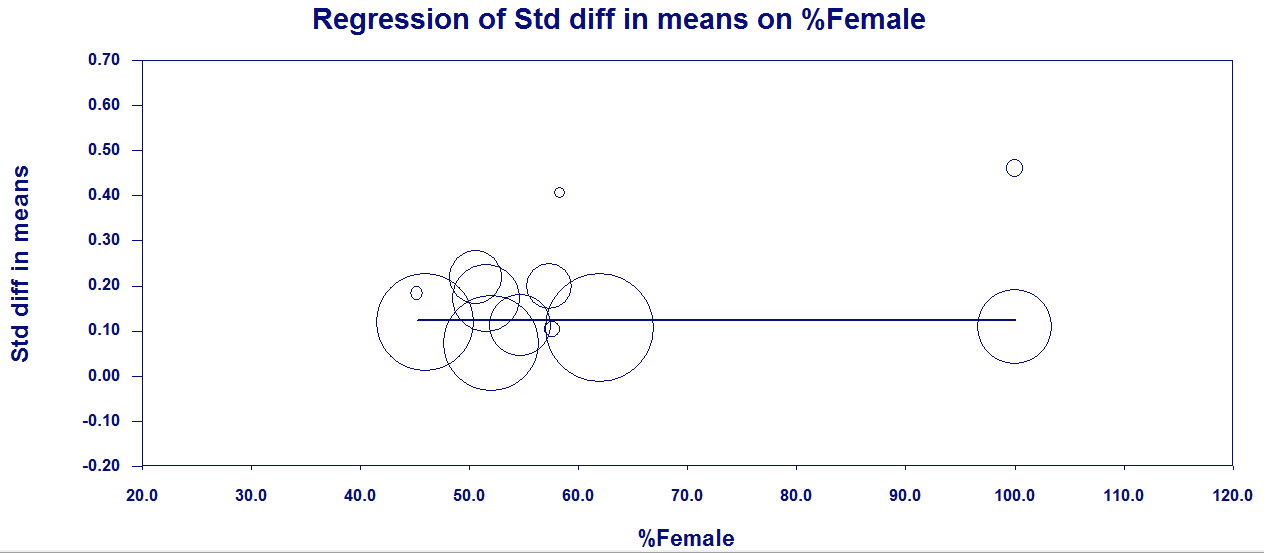


Figure S14. Gait and global cognition - regression of standard difference in means on % female (random-effects model).


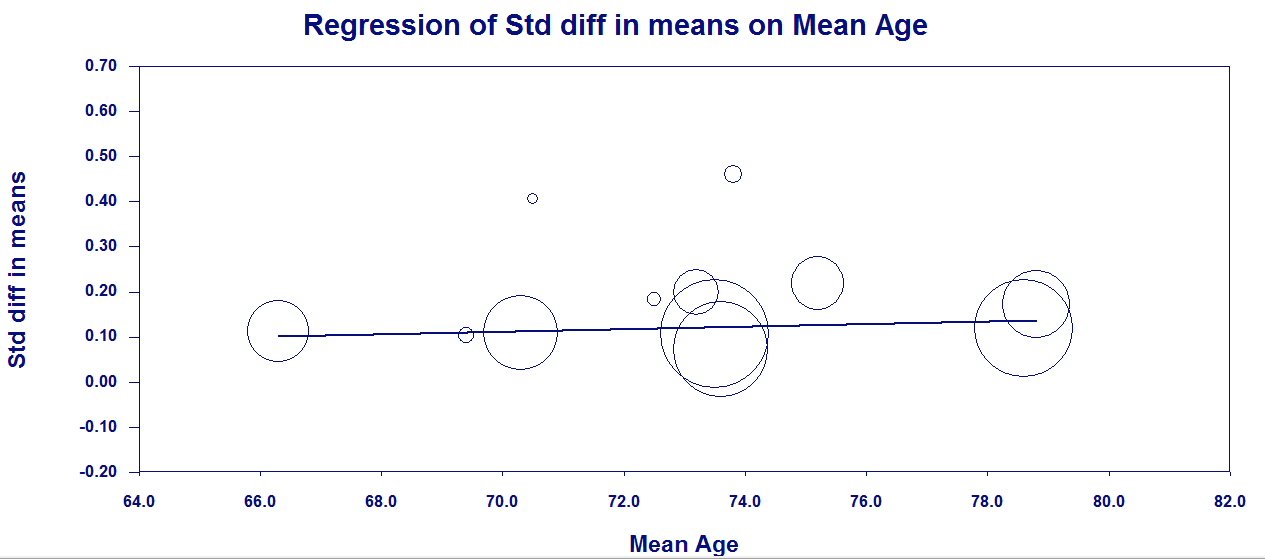


Figure S15. Gait and global cognition - regression of standard difference in means on mean age (random-effects model).


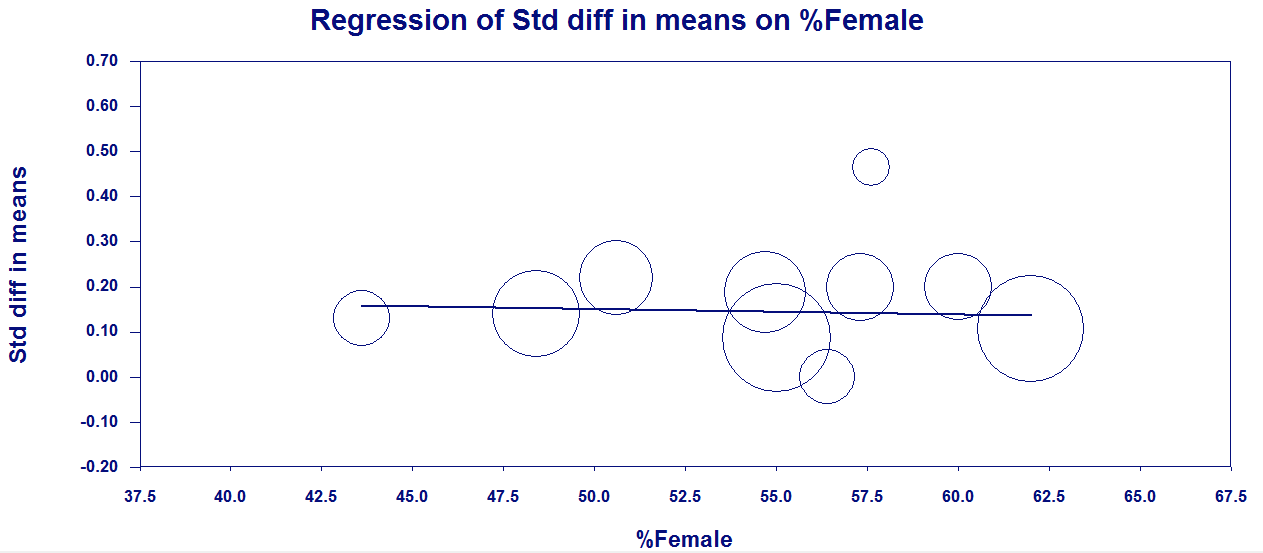


Figure S16. Gait and memory - regression of standard difference in means on % female (random-effects model).


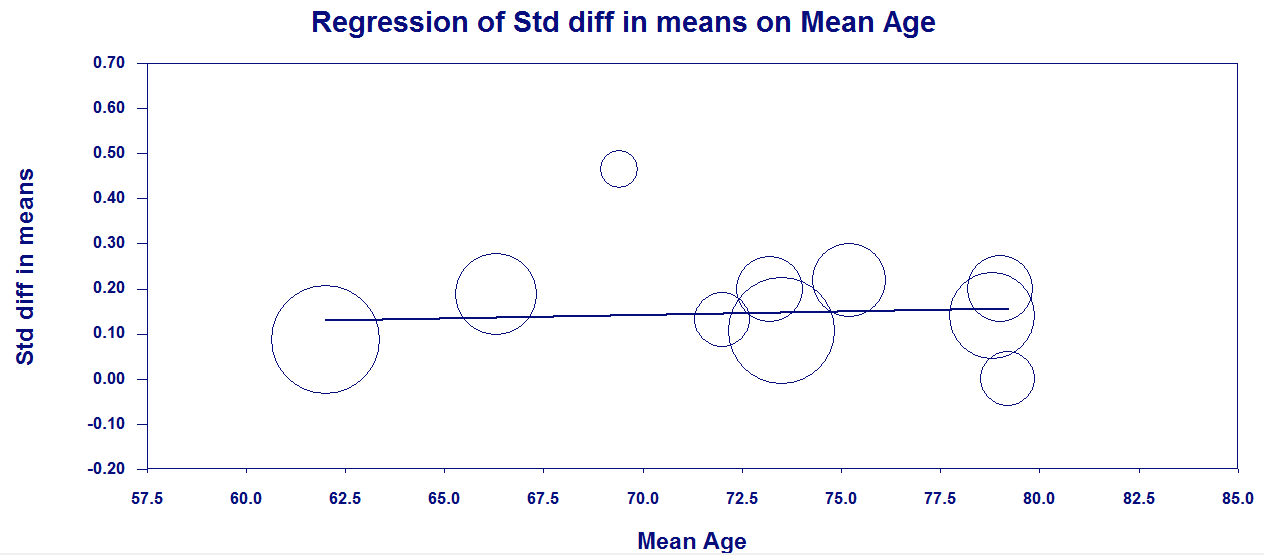
 Figure S17. Gait and memory - regression of standard difference in means on mean age (random-effects model).


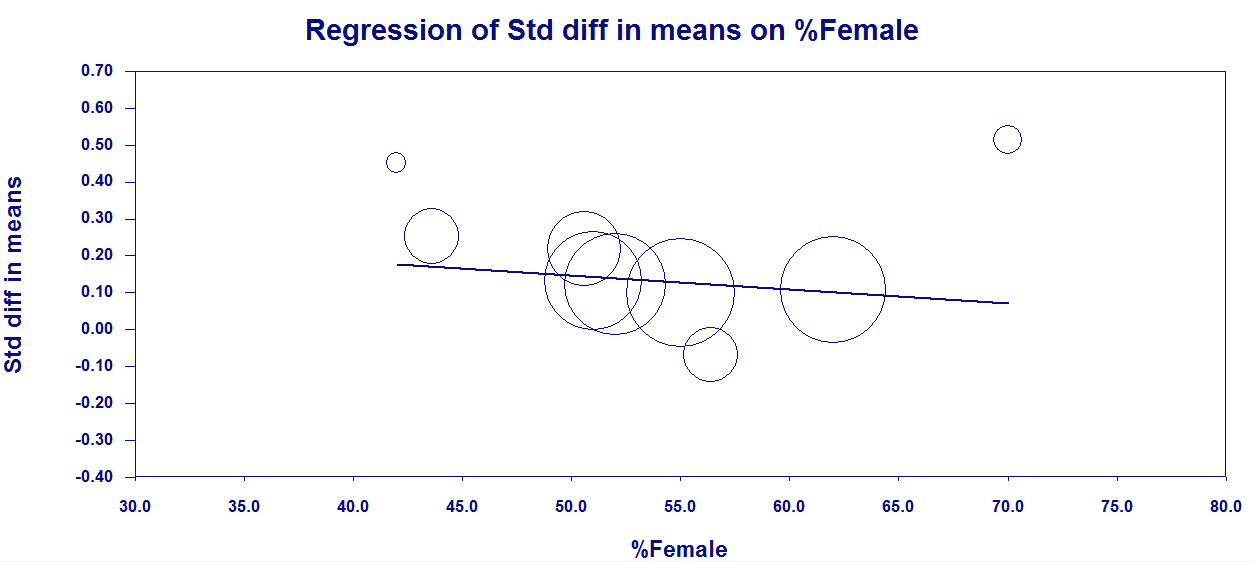


Figure S18. Gait and processing speed - regression of standard difference in means on % female (random-effects model).


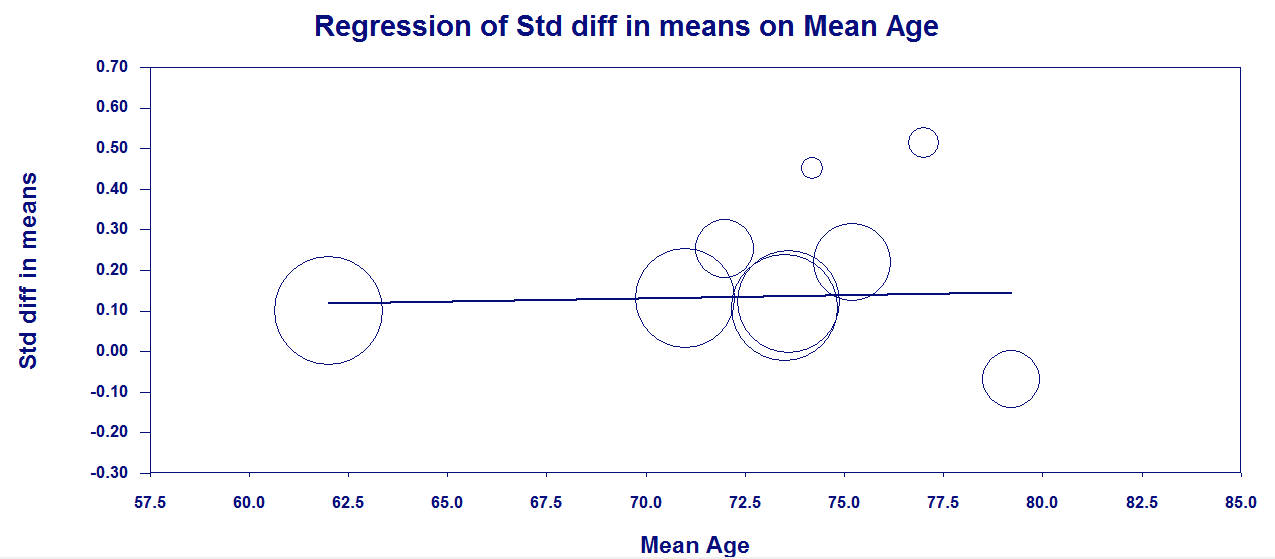


Figure S19. Gait and processing speed - regression of standard difference in means on mean age (random-effects model).
